# PCD’s Midyear Progress Assessment, Journal Rankings, and 20th Anniversary Celebration

**DOI:** 10.5888/pcd21.240312

**Published:** 2024-08-15

**Authors:** Leonard Jack

**Affiliations:** 1 Preventing Chronic Disease, Office of Medicine and Science, National Center for Chronic Disease Prevention and Health Promotion, Centers for Disease Control and Prevention, Atlanta, Georgia

**Figure:**
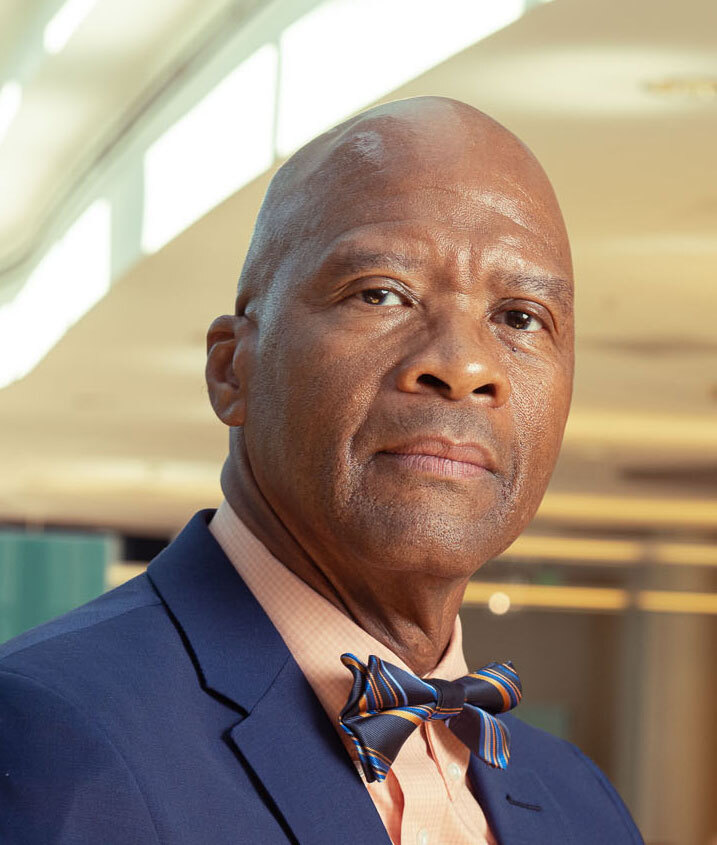
Leonard Jack, Jr, PhD, MSc

*Preventing Chronic Disease* (PCD) remains committed to achieving its mission of serving as an influential journal in the dissemination of proven and promising peer-reviewed public health findings, innovations, and practices and publishing editorial content respected for its integrity and relevance to chronic disease prevention. This Editor in Chief Column provides updates regarding the journal’s latest rankings; recent appointments of volunteer board and committee members; students selected for the Student Scientific Review and Writing Training Committee; recently released and forthcoming collections in 2024; current Calls for Papers; and celebration plans for PCD’s 20th anniversary.

## Journal Impact Factor and Scimago Journal & Country Rank

Because of a change in the way Clarivate calculated the journal impact factor this past year, which collapsed many subcategories into a larger pool under more general subject areas, many journals experienced a decrease in impact factor, and PCD was no exception. Our impact factor decreased from 5.5 in 2022 to 4.4 for 2023. That change is not a reflection of a decrease in the quality of the journal; in fact, the journal continues to improve in the quality, rigor, relevance, and outreach of its content as evidenced by the other primary source of journal rankings: SJR, or Scimago Journal & Country Rank. Those metrics were also recently released, and PCD again made impressive gains in both its domestic and international rankings:

Worldwide Journals in All Categories
  - 2022: Ranked in the top 10% worldwide of 27,955 journals tracked
  - 2023: Ranked in the top 7% worldwide of 29,165 journals tracked
Worldwide Journals in Public Health, Environmental and Occupational Health
  - 2022: Ranked 59th of 608 (top 10%)
  - 2023: Ranked 48th of 656 (top 7%)
Worldwide Open Access Journals in Public Health, Environmental and Occupational Health
  - 2022: Ranked 22nd of 252 (top 9%)
  - 2023: Ranked 21st of 295 (top 7%)
US Journals in Public Health, Environmental and Occupational Health
  - 2022: Ranked 21st of 169 (top 12%)
  - 2023: Ranked 16th of 165 (top 10%)
US Open Access Journals in Public Health, Environmental and Occupational Health
  - 2022: Ranked 3rd of 33 (top 9%)
  - 2023: Ranked 3rd of 36 (top 8%)

PCD’s continued improvement in its domestic and worldwide standings is the result of the tremendous support received from senior leadership in the National Center for Chronic Disease Prevention and Health Promotion at the Centers for Disease Control and Prevention (CDC), along with the journal’s dedicated staff, Associate Editors, Editorial Board, and Statistics Review Committee members.

## New Volunteers on Boards and Committees

The journal’s overall direction and quality assessment of submitted manuscripts are guided by the skillful volunteers who serve on one or more of the journal’s boards and committees. This year, PCD is pleased to announce that 20 talented public health researchers, evaluators, and practitioners were appointed to the journal’s team of Associate Editors (10) and to its Editorial Board (8) and Statistics Review Committee (2). These volunteers will add to the journal’s already strong foundation across several important content areas. Examples of areas of expertise brought to the journal by its recently appointed volunteers include the following:

- Interdisciplinary and intersectional understanding of the relationship between place and health
- Conceptualizing, implementing, and monitoring large-scale studies with community engagement
- Advanced quantitative data analyses and complex data management
- Spatial epidemiologic methods and advanced statistics modeling
- Implementation science and its application in real-world public health and health care settings
- Use of community public health pharmacies to improve chronic disease outcomes
- Business acumen in public health to develop sustainable strategies that support population health
- Screen-time exposures and their effects on nutrition, physical activity, sleep, and chronic disease management and prevention

Please visit the journal’s website to learn more about the background and experience of the journal’s newly appointed Associate Editors (https://www.cdc.gov/pcd/about_the_journal/associate_editors.htm) and Editorial Board members (https://www.cdc.gov/pcd/about_the_journal/editorial_board.htm) and Statistics Review Committee members (https://www.cdc.gov/pcd/about_the_journal/Statistics_Review_Committee.htm) .

## Second Class of Student Scientific Writing and Review Training Committee Members

In March, after careful consideration, 14 students representing all levels of educational training — high school, undergraduate, master’s, doctoral (PhD, DrPH, EdD), medical, and postdoctoral — were selected for the journal’s Student Scientific Writing and Review Training Committee (1). This is the journal’s second class of students selected for this committee. The committee was created last year to build the next generation of public health researchers by providing select students with learning opportunities that will help strengthen their scientific publishing expertise. Students chosen for this committee bring diverse backgrounds and experiences from across the county.

## Collections

In 2023 PCD increased its published collections from 4 to 6, setting a record for the most collections published in a single year. This year PCD is set to break last year’s record, with 7 collections scheduled for publication. The collections group together published articles for rapid dissemination to our readers and address timely public health topics with contributors from around the world. Of the 7 collections scheduled this year; 3 have already been released.

In April, the journal released its first collection of the year, *Mapping Chronic Disease in the United States* (2). This collection features 7 peer-reviewed GIS Snapshots articles highlighting examples of CDC’s National Center for Chronic Disease Prevention and Health Promotion’s use of GIS (Geographic Information System) in preventing and addressing chronic diseases. GIS Snapshots articles are intended to highlight the public health application of maps in a brief format and demonstrate how GIS guides chronic disease prevention and treatment.

PCD released its second collection in May, *From Data to Action: National, State & Local Efforts to End Menthol and Other Flavored Commercial Tobacco Product Use* (3). This collection features 10 articles highlighting the role of public health in reducing tobacco-related diseases and deaths, the use of menthol and other flavored tobacco surveillance data, and state and local activities implemented in these areas.

Most recently, in June, PCD released its third collection, *Policy, Systems, and Environmental Approaches in Chronic Disease Research and Practice* (4). This collection features 7 peer-reviewed articles that explore how research, surveillance, and evaluation can be used to advance the use and application of policy, systems, and environmental approaches in public health.

Later this year, PCD will release collections that address public health actions to reduce the burden of asthma and that address advancing chronic disease data modernization, Other scheduled collections are PCD’s Student Paper Contest, which will include a special section of student essays addressing pressing public health issues, and a special collection dedicated to PCD’s 20th anniversary.

## Current Calls for Papers

Because of the increase in publication of PCD collections, PCD has removed its Calls for Papers (CFPs) from the Announcements web page and created a new special section of the website dedicated to our CFPs. Here’s a list of our current CFPs:

- **2025** **Student Paper Contest**. PCD welcomes submissions from high school, undergraduate, graduate, recent postgraduate, and medical students and residents for PCD’s annual Student Paper Contest using 2 of the journal’s article types: Original Research and GIS Snapshots. PCD is interested in publishing papers relevant to prevention, screening, surveillance, and population-based intervention of chronic diseases, including but not limited to arthritis, asthma, cancer, depression, diabetes, obesity, cardiovascular disease, and COVID-19 and chronic conditions. The journal is also interested in research examining the role that social determinants exact on health, including the less explored determinant of racism (5).
- **Students Have Their Say: Novel Approaches and Solutions to Current and Emerging Public Health Problems**. As part of the Student Paper Contest, this new opportunity allows high school, undergraduate, graduate, recent postgraduate, and medical students and residents to improve their scientific writing skills by serving as lead (first) authors, becoming familiar with a journal’s peer-review process, and receiving feedback from the journal on how to strengthen their submission regardless of whether it is accepted. Students are invited to submit only one type of article: an Essay. Students will use the journal’s essay format to describe persistent or emerging public health challenges with new approaches and solutions (6).
- **Rural Health Disparities: Contemporary Solutions for Persistent Rural Public Health Challenges**. Public health challenges have been documented in rural geographical areas and remain persistent public health, medicine, and health services problems. These challenges include limited health care access, excessive tobacco use in counties with low-income populations, limited physical activity, socioeconomic inequities, behavioral and mental health conditions, and major chronic diseases. PCD encourages the submission of manuscripts covering diverse topics using various article types. We encourage authors to explore the social determinants of health, environmental influences, policy interventions, and community-based initiatives contributing to chronic disease prevention in rural areas (7).
- **Community Engagement and Population Health From Practice to Evaluation**. The complexity of what shapes health makes it important to establish and maintain community and the community’s engagement to increase supportive opportunities to improve health. Several terms and concepts over the years, such as community mobilization, community-based participatory research, and social engagement, have been used to describe the importance of different aspects of community engagement. Regardless of the terminology or concept, working collaboratively with communities to address factors affecting health is central to ameliorating persistent health challenges. For this collection, PCD encourages submissions that reflect on how and where engagement has occurred, including the populations engaged and geographic locations, types of partners involved, community engagement strategies used, and advances in measuring community engagement. PCD encourages the submission of manuscripts covering diverse topics using various article types (8).
- **Screen-Time Effects on Mental Health, Physical Activity, Nutrition, and Sleep Across the Lifespan**. Increased exposure to screen time has become an important public health concern for several reasons. Excessive screen-time exposure has been linked to poor health outcomes such as weight gain, physical inactivity, reduced sleep quality, poor body image perception, poor nutrition, and mental health conditions such as anxiety and depression. Research indicates that the relationship between screen-time use and poor health outcomes exists among children and adults. PCD encourages the submission of manuscripts covering diverse topics using various article types (9).

## PCD Celebrates 20 Years of Publication

PCD was launched in January of 2004, and the journal is marking this important 20-year milestone with a special celebration in October. PCD will host a celebratory event on the campus of CDC, recognizing those responsible for the journal’s conception and implementation, highlighting examples of the journal’s many successes over the years, and discussing important future directions. During the October anniversary celebration, PCD will recognize the journal’s founding Editor in Chief, Dr Lynne S. Wilcox, by introducing an annual PCD “Paper of the Year” in her name and announcing the first winner. Leading up to this event, PCD will share historical milestones and successes on PCD’s website and through social media.

## Conclusion

In just the first 6 months of 2024, PCD has already had an extremely productive year, building on the successes of 2023. We encourage readers to look at what the journal has published so far and to stay connected with us through the remainder of the year and beyond. The journal releases new content every week, which can be accessed by visiting the journal’s website or by subscribing to receive important updates, including a monthly table of contents. In addition, subscribers receive up-to-the-minute updates by following our many social media channels: Facebook, X (formerly Twitter), Instagram, and LinkedIn. The journal’s continuing reputation for quality peer-reviewed content, technical innovation, and integrity and leadership in the field of scholarly publishing is due to the expertise, dedication, and commitment to public health from our authors, volunteers, staff, and CDC leadership. On behalf of the entire PCD team, we say thank you to everyone who has played a major role in positioning the journal for continued success.
